# Seroprevalence and Risk Factors of *Trichinella spiralis* Infection in Blood Donors from Western Romania

**DOI:** 10.3390/medicina58010128

**Published:** 2022-01-15

**Authors:** Radu Pavel, Sorin Ursoniu, Ana Alexandra Paduraru, Rodica Lighezan, Maria Alina Lupu, Tudor Rares Olariu

**Affiliations:** 1 Discipline of Parasitology, Department of Infectious Diseases, Victor Babes University of Medicine and Pharmacy, 300041 Timisoara, Romania; paduraru.ale@gmail.com (A.A.P.); rlighezan@gmail.com (R.L.); mariaalinalupu@gmail.com (M.A.L.); 2 Center for Diagnosis and Study of Parasitic Diseases, Victor Babes University of Medicine and Pharmacy, 300041 Timisoara, Romania; 3 Discipline of Epidemiology, Department of Infectious Diseases, Victor Babes University of Medicine and Pharmacy, 300041 Timisoara, Romania; 4 Discipline of Public Health, Department of Functional Sciences, Center for Translational Research and Systems Medicine, Victor Babes University of Medicine and Pharmacy, 300173 Timisoara, Romania; sursoniu@umft.ro; 5 Clinical Laboratory, Municipal Clinical Emergency Hospital, 300079 Timisoara, Romania; 6 Regional Blood Transfusion Centre, 300737 Timisoara, Romania; 7 Clinical Laboratory, Institute of Cardiovascular Diseases, 300310 Timisoara, Romania

**Keywords:** *Trichinella spiralis*, trichinellosis, Western Romania, risk factors, seroprevalence

## Abstract

*Background and Objectives:* Trichinellosis, a serious and sometimes fatal human disease, is a foodborne zoonotic disease with worldwide distribution caused by parasitic nematodes of the genus *Trichinella*. Humans are infected with *Trichinella* larvae through the ingestion of meat that has not been properly cooked. Romania reported most of the confirmed cases of trichinellosis among the EU countries. The aim of this cross-sectional study was to evaluate, for the first time, the seroprevalence and risk factors of *Trichinella* infection in blood donors from Western Romania. *Materials and Methods:* Serum samples of 1347 consecutive blood donors were investigated using an immunoenzymaticassay (ELISA) for the determination of specific IgG class antibodies against *T.spiralis*. A questionnaire interview was used to obtain information regarding the potential risk factors associated with *T. spiralis* infection. Mantel–Haenszel chi-squared test or the Fisher exact two-tailed test, as appropriate, were used for comparison between *T. spiralis* positive and *T. spiralis* negative blood donors. Student’s *t*-test was used to evaluate differences between means in studied groups and body mass index was calculated by dividing weight in kilograms by height in meters squared. Statistical analysis was performed using Epi Info Version 7.2 and Stata 16.1. *Results: T. spiralis* IgG antibodies were detected in 2.00% (27) of 1347 consecutive blood donors. Eating raw and/or undercooked meat, from pigs or wild boars, was found to be the main risk factor (*p* < 0.001). Strong alcoholic drink consumption was highly associated with *T. spiralis* infection (*p* = 0.009). *Trichinella* seroprevalence was higher among rural residents and males. Subjects identified as *Trichinella* seropositive were not previously diagnosed and have not been treated for *Trichinella* infection with any specific therapy. *Conclusions:* The demonstration of *T. spiralis* antibodies in healthy blood donors suggests that *Trichinella* infection may be detected in asymptomatic individuals that were not previously diagnosed with this zoonosis.

## 1. Introduction

Trichinellosis, a serious and sometimes fatal human disease, is a foodborne zoonotic disease with worldwide distribution [1] caused by parasitic nematodes of the genus *Trichinella* [2]. Humans are infected with *Trichinella* larvae through the ingestion of meat that has not been properly cooked. It can cause acute and chronic illness [3] and has historically been associated with consumption of pork. Wild boar meat is currently the second most important source of human trichinellosis and has been responsible for many human outbreaks reported in recent years in Europe [1].

The life cycle of *Trichinella* can be divided into two phases: an (intestinal) enteral phase, and a muscular (parenteral or systemic) phase [2]. In the intestinal phase, the larvae are released in the stomach, then penetrate the mucosa of the small intestine and mature into adult worms [2]. In the second or muscular phase, new born larvae migrate throughout the body until they reach the cells of the striated skeletal muscles [2]. Greater priority should be given to this zoonosis because of its health and economic impact, particularly in resource-poor countries [4]. However, estimation of the impact of trichinellosis in nonindustrialized countries with reference to health, social, and economic costs is difficult [5]. The number of infections is underreported in many countries due to the lack of appropriate serological tests and a lack of knowledge of this parasitic disease on the part of physicians [4].The severity of trichinellosis depends mainly on the number of larvae ingested (the infecting dose); the frequency of consumption of infected meat; and how the meat was cooked or treated [4]. 

The humoral immune response leads to the production of parasite-specific antibodies which have a great diagnostic value and the persistence of IgG antibodies may last for many years, even in benign or asymptomatic cases [4].

Few studies assessed the seroprevalence of trichinellosis in the human population [6,7,8,9]. The vast majority of published reports evaluated trichinellosis in hospitalized patients or described outbreaks caused by *Trichinella* spp. [10,11,12,13,14,15]. In Europe, Romania reported most of the confirmed cases of trichinellosis. However, since 2016 it has been overtaken by Bulgaria and in 2020 even by Italy according to the ECDC database [16]. In Romania, the reported incidence (cases per 100,000 inhabitants), over the last five years varied from 0.24 in 2017 to 0.02 in 2020 [16].

The prevalence of *Trichinella* infection in asymptomatic Romanian individuals is unknown and no scientific reports were published in the international literature regarding risk factors for *Trichinella* infection in Romania. Therefore, we evaluated for the first time the seroprevalence and risk factors of *Trichinella* infectionin blood donors from Western Romania, a well-known endemic region [11,12].

## 2. Materials and Methods

We conducted a cross-sectional study and investigated sera for *T. spiralis* antibodies of 1347 consecutive blood donors attending the Regional Blood Transfusion Center in Timișoara, Romania. Serum samples were tested at the Center for Diagnosis and Study of Parasitic Diseases, at the “Victor Babeș” University of Medicine and Pharmacy Timișoara laboratory. All blood donors complied with the donation eligibility criteria set by the Romanian Ministry of Health [17]. Blood donors were residing in four counties located in Western Romania (the counties of Timiș, Caraș-Severin, Hunedoara, and Arad), with a total population of 1,828,313 inhabitants. Participants were grouped into four age categories as follows: 18–29 years, 30–39 years, 40–49 years, and 50–63 years.

Serum samples collected between 19 November 2018 and 21 December 2018 were obtained by venipuncture and then kept at −20 °C until use.

Serum samples were tested for *T. spiralis* antibodies using the NovaLisa IgG enzyme immunoassays (ELISA) (NovaTec ImmunodiagnosticaGmbH, Dietzenbach, Germany), according to the manufacturer’s instructions. This ELISA technique is a qualitative Immunoenzimatic assay useful for the determination of specific IgG class antibodies against *Trichinella spiralis* in human serum.

The interpretation of NovaLisa tests results was based on the manufacturer’s criteria for *T. spiralis* IgG antibodies as follows: <9 NTU (NovaTec Units) negative; 9–11 NTU equivocal; >11 NTU positive. For the purpose of this study, equivocal results were considered negative [18]. A questionnaire interview for blood donors was applied to obtain information regarding the main risk factors associated with *T. spiralis* infection, including a history of eating raw or undercooked meat from pork or wild boar. Blood donors were also asked questions regarding age, gender, body weight, height, residence area, household ownership, and pig husbandry. Additional data were acquired regarding employment, occupation, educational level (primary education, lower secondary education, vocational school, high school, post-secondary school, bachelor studies/postgraduate), alcohol consumption, history of being diagnosed with *Trichinella* infection, and if they have been treated for *Trichinella* infection with specific therapy.

Data were compiled in a Microsoft Excel database, version 2011 (Microsoft Corp., Redmond, WA, USA). Descriptive statistics (means, standard deviations, and proportions) were calculated. Bartlett’s test was used to determine whether the variables were distributed normally. Student’s *t*-test was used to evaluate differences between means in studied groups. Mantel–Haenszel chi-squared test or the Fisher exact test two-tailed, as appropriate, were used to compare frequencies distribution between *T. spiralis* positive and *T. spiralis* negative blood donors. The body mass index was calculated by dividing weight in kilograms by height in meters squared. Odds ratios (ORs) and their 95% confidence intervals (95% CI) were calculated for all variables to identify risk factors associated with *T. spiralis* IgG antibodies. Statistical analysis was performed using Epi Info Version 7.2 (CDC, Atlanta, GA, USA) and Stata 16.1 (Statacorp, College Station, TX, USA). A probability level of *p* < 0.05 was considered to indicate statistical significance.

This study was approved by the “Victor Babeș” University Ethics Committee and informed consent was obtained from all blood donors. Participation in the study was voluntary and individuals were informed about the purpose of this study.

## 3. Results

Among 1347 blood donors, aged 18–63 years (mean age = 33.58; SD ± 10.90), *T. spiralis* IgG antibodies were detected in 2.00% (27) as follows: 1.42% (8/563) in those aged ≤ 29 years, 1.90% (7/369) in those aged 30–39 years, 2.13% (6/281) in those aged 40–49 years, 4.48% (6/134) in those aged ≥50 years, and their presence tended to increase with age (Table 1). No statistically significant difference was found between the average age of *Trichinella* seropositive subjects and *Trichinella* seronegative subjects (*p* = 0.16) (Table 1).

*T. spiralis* seropositivity was 2.52% in males (19/755) compared to 1.35% in females (8/592) (*p* = 0.13) (Table 1). Also, *Trichinella* seropositive males were significantly older (range = 22–63) (mean = 40.21; SD ± 12.95) than *Trichinella* seropositive females, (range = 19–43) (mean=29.75; SD ± 9.82); (*p* = 0.03) (data not shown).

The prevalence of IgG antibodies was higher in blood donors from rural areas 2.45% (9/368) than in those from urban areas 1.84% (18/979) but was not associated with *Trichinella* infection (*p* = 0.47) (Table 1).

In terms of body mass index (BMI), *T. spiralis* IgG antibodies in seropositive subjects was distributed as follows: 14.29% (1/7) underweight (BMI below 18.5), 1.51% (9/596) normal or healthy weight (BMI 18.5–24.9), 1.89% (9/475) overweight (BMI 25.0–29.9), and 2.97% (8/269) obese (BMI 30.0 and above) (Table 1). Underweight body mass index subjects were found to be associated with *T. spiralis* infection (*p* = 0.008) (Table 1). No significant difference was found between average body mass index for *Trichinella* seropositive subjects and *Trichinella* seronegative subjects (*p* = 0.48) (Table 1).

Data regarding value of eosinophil count from laboratory investigation results, initially performed at Regional Blood Transfusion Center in Timișoara, after blood donation, were available for 15 (55.5%) of the 27 *Trichinella* seropositive subjects. Five of them (33.3%) had higher values of eosinophil count and 10 (66.6%) had normal values (1–6%) [19] (data not shown).

*Trichinella* seroprevalence varied according to the educational level of participants: bachelor studies/postgraduate 2.51% (16/638), post-secondary school 3.85% (2/52), high school 0.66% (3/453), vocational school 3.33% (4/120), lower secondary education 1.85% (1/54), and primary education 3.33% (1/30) (Table 1). Our observations showed no association between employment category and *T. spiralis* IgG antibodies (Table 1). Owning a household 2.78% (11/395) and pig husbandry 1.65% (2/121), were not associated with *T. spiralis* IgG antibodies (Table 2).

Eating raw and/or undercooked meat 4.04% (23/569; *p* < 0.001), from pork 3.97% (16/403; *p* < 0.001) or wild boar 6.44% (21/326; *p* < 0.001) was the highest risk factor for acquiring *Trichinella* infection (Table 2).

Strong alcoholic drink consumption 4.86% (7/144) was highly associated with *T. spiralis* IgG antibodies seropositivity (*p* = 0.009) (Table 2).

*Trichinella* seropositive subjects declared that they had not been diagnosed (81.48%; 22/27) or did not know that they had *Trichinella* infection (18.51%; 5/27) and none of them had been treated for *Trichinella* infection with specific therapy (100%; 27/27) (data not shown).

## 4. Discussion

The present study revealed that *T. spiralis* seroprevalence in blood donors from Western Romania was 2.00%. This is similar with the 2.2% rate reported in Republic of Sakha (Yakutia) a federal Russian republic [6], and lower than the rates reported in Estonia 3.1% [7], China 3.19% [8] and Chukotka Autonomous Okrug, Far Eastern District of the Russian Federation 24.3% [9].

The notification rate has been steadily decreasing at a national level according to the ECDC database [16] and our estimated seroprevalence (lower compared to other reported rates), is in accordance with the declining trend of such incidence.

Our results also revealed that *Trichinella* seroprevalence tended to increase with age and this observation is in accordance with results reported by Cui et al. [8].

In terms of gender, some studies showed that trichinellosis infections occur almost equally among both sexes [20] while other findings indicate a higher prevalence in females [21,22]. Our observations suggest that male subjects may be more exposed to *T. spiralis* infection than female subjects.

It is known that high eosinophilia is one of the most frequently observed laboratory features, it regresses slowly and can remain at lower levels for several weeks to months [2]. We found that eosinophil count data available for our seropositive subjects showed that two-thirds of participants had normal values of eosinophil count. This suggests that most of the infected subjects were identified in the convalescent stage of *Trichinella* infection.

Results of the present study suggest that blood donors with post-secondary school, vocational school and primary education may have more frequently *T. spiralis* IgG antibodies compared to subjects with different level of education. Other studies described seropositivity to *T. spiralis* in individuals with an incomplete elementary education (primary education) [23].

Our findings indicate that consumption of raw and/or undercooked meat from pigs or wild boars was the highest risk factor for acquiring *Trichinella* infection, similar as described by others [8,23].

Results of this study also suggest that strong alcoholic drinks consumption may be associated with *T. spiralis* infection and that could be an adult orientated food behavior, culture driven with improperly cooked meat consumption [20]. This highly associated alcohol consumption with *T. spiralis* IgG seropositive subjects could be an explanation for the asymptomatic clinical form of *Trichinella* infection, given that alcohol could increase the resistance to the infection [2]. Moreover, the *Trichinella* seropositive subjects, declared that they had not been diagnosed or did not know they had *Trichinella* infection and all of them had been not treated for *Trichinella* infection with specific therapy.

According to the Commission Regulation 2015/1375, carcasses of domestic pigs and wild boar that are intended for human consumption in the EU market should be systematically tested for *Trichinella* at slaughter as part of the meat inspection process for the presence of the parasite larvae in the muscles [24]. Neither domestic pigs nor wild boars slaughtered for own consumption are covered by the above-mentioned regulation [24].

This study has limitations. Blood donors, as a specific subgroup of the adult general population, includes healthy individuals and may present different demographic characteristics in terms of age, area of residence, or gender [25]. Children, teenagers, or individuals older than 64 years were not evaluated in the present study and the number of individuals from urban area was higher than those from rural area. In addition, the seroprevalence of *Trichinella* infection in humans can vary widely depending on the population studied and may be higher in subjects with history of eating raw or semi-cooked meat (such as pork or wild boar) [8]. Due to the relatively low number of positive cases identified in this survey, further epidemiological studies that assess the potential risk factors for *Trichinella* infection should be performed on larger groups of subjects.

## 5. Conclusions

Testing for *T. spiralis* antibodies in our studied group indicated a low rate of *T. spiralis* infection in Western Romania. However, this seroprevalence suggests that *Trichinella* infectionmay be detected in healthy and asymptomatic individuals that were not previously diagnosed with this zoonosis. Consumption of raw and/or undercooked meat, from pigs or wild boars, was found to be the main risk factor for acquiring *Trichinella* infection. Therefore, preventive measures and education of the population should be maintained and permanently improved.

## Figures and Tables

**Table 1:** Baseline characteristics and descriptive statistics in blood donors ascertained by questionnaire.

Descriptive Statistics	Variables	*Trichinella* Seropositive (*n* = 27)	*Trichinella* Seronegative (*n* = 1320)	(95% CI)			
				OR	Lower Limit	Upper Limit	*p*-Value
Age (years) (33.58 ± 10.90) ^1^		(37.11 ± 12.87) ^1^	(33.50 ± 10.85) ^1^	(0.16) ^2^			
Age categories	≤29	8	555		1 (Ref.)		-
	30–39	7	362	1.34	0.48	3.73	0.57
	40–49	6	275	1.51	0.52	4.40	0.44
	≥50	6	128	3.25	1.10	9.53	0.03
Gender	Males	19	736	1.88	0.81	4.33	0.13
Residence area	Rural	9	359	1.33	0.59	3.00	0.47
Height (cm) (173.02 ± 9.24) ^1^		(176.51 ± 7.27) ^1^	(172.95 ± 9.27) ^1^	(0.01) ^2^			
Weight (Kg) (79.29 ± 17.03) ^1^		(84.74 ± 18.02) ^1^	(79.18 ± 17.00) ^1^	(0.12) ^2^			
Body mass index (BMI) (26.40 ± 4.92) ^1^		(27.09 ± 5.08) ^1^	(26.39 ± 4.92) ^1^	(0.48) ^2^			
Body mass index	Underweight	1	6	10.87	1.16	101.26	0.008
	Normal	9	587		1 (Ref.)		-
	Overweight	9	466	1.25	0.49	3.20	0.62
	Obese	8	261	1.99	0.76	5.24	0.15
Education level	Bachelor studies/Postgraduate	16	622		1 (Ref.)		
	Post-secondary school	2	50	1.55	0.34	6.96	0.56
	High school	3	450	0.25	0.07	0.89	0.02
	Vocational School	4	116	1.34	0.43	4.08	0.60
	Lower secondary education	1	53	0.73	0.09	5.64	0.76
	Primary education	1	29	1.34	0.17	10.47	0.77
Employment	employed	18	851		1 (Ref.)		
	unemployed	4	163	1.16	0.38	3.47	0.79
	student	5	286	0.82	0.3	2.24	0.7
	retired	0	20	NA	-	-	0.51

^1^ Values presented in parentheses are mean value ± standard deviation. ^2^ Student’s *t*-test was used to evaluate differences between means; *p* value presented in parentheses CI, confidence interval; Ref., reference; OR, Odds ratio.

**Table 2:** Risk factors for *Trichinella* infectionin blood donors ascertained by questionnaire.

Potential Risk Factor	Variables	*Trichinella* Seropositive (*n* = 27)	*Trichinella* Seronegative (*n* = 1320)	(95% CI)			
				OR	Lower Limit	Upper Limit	*p*-Value
Occupation	Owning a household	11	384	1.67	0.77	3.64	0.188
	Pig husbandry	2	119	0.80	0.18	3.45	1
Food habits	Eating raw and/or undercooked meat (yes vs. no)	23	546	8.15	2.80	23.70	<0.001
	Eating raw and/or undercooked pork meat	16	387	3.50	1.61	7.62	<0.001
	Eating raw and/or undercooked wild boar meat	21	305	11.64	4.65	29.11	<0.001
	Strong alcoholic drink consumption	7	137	3.02	1.25	7.27	0.009

CI, confidence interval; NA, not applicable; Ref., reference; OR, Odds ratio.

## Data Availability

Data available on request.
